# Beyond de-foaming: the effects of antifoams on bioprocess productivity

**DOI:** 10.5936/csbj.201210014

**Published:** 2012-12-01

**Authors:** Sarah J Routledge

**Affiliations:** a School of Life and Health Sciences, Aston University, Aston Triangle, Birmingham B4 7ET, United Kingdom

**Keywords:** Recombinant protein, optimization, oxygen transfer, *kLa*, *P. pastoris*

## Abstract

Antifoams are often added to bioprocesses with little knowledge of their impact on the cells or product. However, it is known that certain antifoams can affect the growth rates of both prokaryotic and eukaryotic organisms in addition to changing surface properties such as lipid content, resulting in changes to permeability. This in turn can be beneficial to a recombinant protein production system for soluble proteins, as has been demonstrated by increased secretion of α-amylase and GFP, or achievement of greater yields of protein due to increased biomass. However, in some cases, certain concentrations of antifoams appear to have a detrimental effect upon cells and protein production, and the effects vary depending upon the protein being expressed. These findings emphasise the importance of optimising and understanding antifoam addition to bioprocesses.

## Foaming in bioprocesses

Foam occurs in bioprocesses due to the introduction of gases into the culture medium, and is further stabilised by proteins produced by organisms in the culture[1]. Foam is made up of liquid lamellas which are full of gas. Foams with high liquid content are unstable, while dry polyhedric foams are more stable and usually formed due to mechanical stresses[2]; both types can be found in bioprocesses. Examples of undesired foam formation is seen in bioprocesses used for paper, food, beverage and drug production such as the synthesis of antibiotics[3]. Unwanted foaming can also occur during water purification, blood transfusions, and in the dyeing of fabrics[3, 4]. In this review, I focus on the foaming that typically occurs in bioprocesses producing recombinant proteins.

The production of recombinant proteins on large scales is essential for the development of drugs as well as the engineering of antibodies[5], the identification of functions and interactions of proteins[6] and also in the production of enzymes[7]. Valuable proteins such as insulin[8] and human growth hormone[9] have been produced recombinantly on an industrial scale in bioreactors and have enabled treatment and understanding of many diseases. In these formats, foaming is a problem that is particularly acute due to gassing used to maintain appropriate dissolved oxygen (DO) concentrations. Foaming can lead to reduced process productivity since bursting bubbles can damage proteins[10], result in loss of sterility if the foam escapes the bioreactor[11] or lead to over-pressure if a foam-out blocks an exit filter. To prevent the formation of foam, mechanical foam breakers, ultrasound or, most often, the addition of chemical antifoaming agents (or “antifoams”)[11] are routinely employed in bioreactors and large shake flasks. There is a well-established literature on antifoams, highlighting their importance in bioprocesses, but relatively little information on how they affect the biology of the process itself[11]. In this review, the effects of antifoams, both positive and negative, on bioprocess productivity are discussed.

## Antifoams

Antifoams can be classified as either hydrophobic solids dispersed in carrier oil, aqueous suspensions/emulsions, liquid single components or solids[12–14] and may contain surfactants[15]. Many antifoaming agents are commercially available, with 43 currently being sold by Sigma-Aldrich alone. While little information is routinely given about the composition of antifoaming agents, their specific defoaming properties have been thoroughly investigated. These include their effects on foam height with time, their influence on the volumetric oxygen mass transfer coefficient (*k*_*L*_*a*) of the system, their gas hold-up characteristics and their globule size and distribution in relation to their action upon foams. Much of the literature available on antifoams in bioprocesses in bioreactors documents their effects upon the DO and the volumetric mass oxygen transfer coefficient (*k*_*L*_*a*) in a system[16–24], rather than upon cells and recombinant proteins.

Antifoams can be split into two categories of fast and slow antifoams, depending on their mechanism of foam destruction: slow antifoams are often oils which destroy foam over a longer period of time, while fast antifoams, are usually mixed agents which enter the foam film[25]. Some simple methods of determining the ability of antifoams to reduce foam are the Bartsch shaking test[26] and the Ross-Miles pouring test[27].

## De-foaming mechanisms

**Figure 1:**
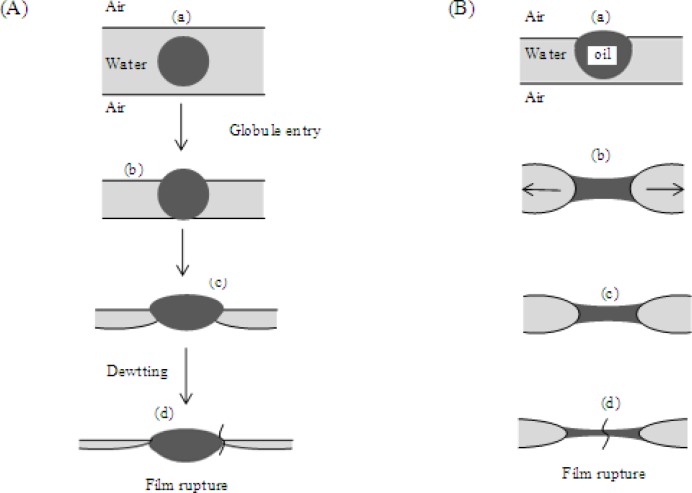
**Bridging-dewetting and bridging-stretching antifoam mechanisms**. (A) Bridging-dewetting, where an oil drop becomes a lens, rupturing the film, and (B) bridging-stretching where the oil particle bridges the foam film surface forming an oil bridge; this stretches forming an unstable film, eventually rupturing the foam. Adapted from Denkov and Marinova 2006[3].

Several mechanisms explaining the action of antifoams have been suggested which include bridging-dewetting, spreading fluid entrainment and bridging-stretching[25]. For oil-based antifoams, bridging-dewetting and bridging-stretching mechanisms are known to occur and are illustrated in Fig 1. Bridging-dewetting (Fig 1A) occurs when an oil drop enters the surface of the foam film and is deformed into a lens shape (Fig 1A (c)). When the film thins, the lens enters the opposite surface of the foam film and forms a bridge. The film is dewetted away from the oil bridge by capillary forces causing the film to rupture (Fig 1A (d)). With bridging stretching (Fig 1B), the oil particle bridges the foam film surface (Fig 1B (a) and (b)). This leads to the formation of an oil bridge which stretches over time, becoming an unstable film that ruptures at the thinnest region so that the entire foam structure is destroyed (Fig 1B (c) and (d))[3, 28]. Mixed agents enter the foam and destroy it in this manner (Fig 1B)[25].

## Antifoams and oxygen transfer

In order to grow, aerobic organisms require a sufficient concentration of dissolved oxygen in the medium. The oxygen transfer rate (OTR) depends upon the *k*_*L*_*a* and upon C_l,8_ - C_l_, where C_l_ is the dissolved oxygen concentration and C_l,8_ is the oxygen saturation concentration in the liquid phase at the gas-liquid interface[29]. The *k*_*L*_*a* is a measure of how much oxygen is transferred into the medium over a certain amount of time[24]. The *k*_*L*_*a* of a system can be influenced by several factors such as properties of the medium like viscosity, the presence of organisms and their by-products. Additions to the medium such as antifoams also have an effect[23, 24]. It has been observed that low concentrations of antifoam can reduce the *k*_*L*_*a* but at higher concentrations the *k*_*L*_*a* may rise[20, 22]. To ensure optimum oxygen transfer within a system, the effect of differing concentrations of the antifoam to be used should be assessed, although this is not typically done. Changes to the *k*_*L*_*a* can be due to effects on *kl* (m/s) and on *a* (specific surface area m^-1^)[20, 30]. It has been suggested that antifoams enhance bubble coalescence and increase bubble size leading to a reduction in the specific surface area therefore lowering *k*_*L*_*a*[11, 16, 17, 20, 30]. However it has also been observed previously that the *k*_*L*_*a* rises at higher concentrations of antifoam agents. This may be due to bubble coalescence reducing the surface tension, which then leads to decreasing bubble size and the *k*_*L*_*a* rises again. Secondly it is possible that antifoams accumulate oxygen from rising bubbles as they have good oxygen solubility, and release it to the aqueous phase. Bubbles bursting at the surface also disperse small drops of the antifoam causing more oxygen to be released[20, 22]. In the case of oils which have a greater oxygen solubility than water, oil droplets may increase oxygen permeability in the water boundary layer of the gaseous dispersion[31]. The ability of antifoams to reduce *k*_*L*_ has been suggested to be less for bubble swarms than for a single bubble[21]. It is also possible that surfactants can lead to rippling or eddying which influences the *k*_*L*_*a*. *k*_*L*_ has not been found to be greatly affected by antifoam agents, with the main effect being upon *a*[23].

In bioprocesses both positive and negative effects of antifoams upon oxygen transfer have been observed, for example a silicone-based antifoam negatively affected the mass transfer coefficient, gas hold up and gas velocity within the media[16]. However it was found by Koch *et al* that antifoams without silicone oil did not greatly affect the oxygen transfer rate, whereas those containing silicone oil had a significant effect at the beginning of the process, which decreased over the duration[19]. Our research has demonstrated that in shake flasks the *k*_*L*_*a* was higher at concentrations of 0.4% v/v to 0.6% v/v and decreased with increasing concentration up to 1% v/v. Additionally, DO in shake flask cultures of *P. pastoris* was unaffected by the presence of antifoam, suggesting that any changes to *k*_*L*_*a* were not great enough to influence the DO in the culture[32]. These DO measurements have been performed in various growth media in both the absence and presence of cultures of prokaryotic and eukaryotic microbes[1, 11, 13, 16, 18, 19, 25]. In contrast, literature on the biological effects of antifoams on recombinant protein production by microbial host cells is more limited, suggesting that this area is not routinely considered.

## Antifoams and recombinant protein production in prokaryotes

A study by Koch and colleagues investigated the effects of several antifoams upon foam destruction as well as upon protein production. The agents tested included; silicone oil (S184); polypropylene glycol (PPG) (SLM54474); silicone oil/PPG mixture (VP1133); and an emulsion containing 10% S184 (SE9). The antifoams were added at various concentrations to *E. coli* K12 cultures producing β-galactosidase fusion protein. It was found that at 1000 ppm of PPG/silicone oil mixture, 555 ppm of emulsion and increasing concentrations of PPG, the specific growth rate of the cells was reduced compared to starting concentrations of under 125 ppm. The other antifoams at increasing concentrations appeared to have no significant effect upon the growth of the cells, although the highest growth rates were achieved in the presence of the emulsion. The mass of the cells grown in the presence of the emulsion was also approximately double that of the cells with the other antifoams. The volumetric and specific product activity of β-galactosidase fusion protein increased with increasing concentrations of PPG and PPG/silicone oil mixtures, while decreased with increasing S184 concentration. This study highlights the range of effects different antifoam compositions could exert upon a culture and also that the concentration applied should be considered, although possible mechanisms of action of the antifoams were not explained[19].

The influence of PEGs of two different molecular weights and various concentrations upon *Bacillus subtilis* and *Bacillus amyloliquefaciens* producing α-amylase has been studied by Andersson *et al*. The *Bacillus* species were cultured in a two-phase aqueous system composed of PEG600 at 8% w/v and 20% w/v in addition to PEG3350 at 5% w/v, 9% w/v and 7% w/v. The production of α-amylase by *B. subtilis* was doubled in the presence of PEG600 at 8% combined with 5% PEG3350, but decreased with 9% PEG3350 alone. An increase in production was also reported with 20% PEG600 for *B. subtilis*, but resulted in a decrease for *B. amyloliquefaciens cultures*. A change in the morphology of the cells was also observed using an aqueous two-phase system, and the PEGs appeared to interact with the cell walls causing the *B. subtilis* cells to become more hydrophilic and *B. amyloliquefaciens* cells to become more hydrophobic and partition to different phases. The difference in the effect of the PEG upon the two organisms was speculated to be due to the influence upon different cell wall or membrane compositions, however no definitive conclusions could be drawn[33].

An investigation conducted by Rao *et al* focused upon the effects of surfactants such as Tween which are not typically used as antifoaming agents upon *Geobacillus thermoleovorans* secreting α-amylase in 250 mL shake flasks. However, the effect of various molecular weights of PEG were also studied, and it was found that PEG with weights above 4000 at 0.5% w/v caused a decrease in specific enzyme activity but increased titres of enzyme. PEGs with lower molecular weights resulted in production of enzyme with greater specific activity than those of higher weights, but slightly lower enzyme titres. Above 0.5% PEG, α-amylase production decreased. The authors suggested that this increase in α-amylase titres could be due to alteration of the membrane phospholipids of the organism, aiding secretion of the enzyme[34].

Overall, antifoams appear affect the growth and recombinant protein production of prokaryotic cultures differently, depending upon the type and concentration used.

## Antifoams and recombinant protein production in eukaryotes

*Schizosaccharomyces pombe* cultures secreting human transferrin (hTF) were grown in the presence of PEG8000 as well as various surfactants. PEG8000 at 0.1% improved the secretion of hTF, however at 1% a growth defect was observed. The data implied that the PEG had altered the phospholipid composition of the cell resulting in an increase in hTF at low concentrations[35].

Both *Saccharomyces cerevisiae* and *P. pastoris* expressing a recombinant Fc fusion protein in shake flask cultures were influenced by the type of antifoam, the concentration, and the combination of antifoam and medium used. Increasing concentrations of alkoxylated fatty acid ester on a vegetable base (J673A) ranging from 0% to 8% v/v added to *P. pastoris* YPD cultures resulted in increase in cells as determined by optical density. Increasing polyalkylene glycol (SB2121) added at 0% to 8% v/v to *S. cerevisiae* SD-URA cultures caused a decrease in cells. It was also found that silicone polymer (Antifoam C) addition of up to 8% v/v to *S. cerevisiae* cultures in YPD medium did not affect the cells. Concentrations of antifoam above 1% appeared to result in a decrease in recombinant protein production although certain agents at higher concentrations improved cell growth.

**Figure 2:**
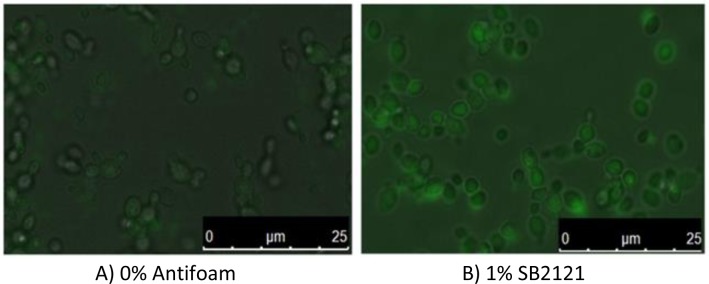
Fluorescence microscopy images of *P. pastoris* producing GFP with and without antifoam viewed under a fluorescence microscope at 100 х magnification. Both intracellular and extracellular GFP is observed. A Leica Microsystems DMI4000B microscope with a Leica CCD camera and Leica application suite AF software were used.

We have recently reported the effects of five antifoams upon recombinant green fluorescent protein (GFP) production by *Pichia pastoris*. Addition of concentrations between 0% v/v and 1% v/v of a 30% emulsion of silicone polymer (Antifoam A), 30% emulsion of silicone polymer with different non-ionic emulsifiers to Antifoam A (Antifoam C), an alkoxylated fatty acid ester on a vegetable base (J673A), a polypropylene glycol (P2000) or polyalkylene glycol (SB2121) to shake-flask cultures of *P. pastoris* increased the total yield of recombinant GFP in the culture medium. In the case of cultures containing P2000, SB2121 and J673A, the yield was almost doubled. The cultures at the optimum concentrations of antifoam were imaged using a fluorescence microscope (Fig 2).) and highlight the differences in GFP produced by the cultures. When normalized to the culture density, the specific yield of GFP (µg OD_595_^-1^) was only increased for Antifoam A, Antifoam C and J673A. This suggested that the enhancements in total yield due to P2000 or SB2121 addition might be attributable to changed growth characteristics of the cells, and these two antifoams were found to the increase culture density. The growth rates for the log phase cultures in the presence of the antifoams suggest that cultures containing 0.8% Antifoam C had the slowest growth, whereas the highest yielding antifoams, J673A, P2000 and SB2121 also had higher growth rates, with J673A growing the fastest at µ = 0.19 h^-1^ compared to the control where µ = 0.13 h^-1^ (unpublished data). We found that the antifoams did not affect the viability of the cells, measured by propidium iodide exclusion and flow cytometry. There was no correlation between total yield, specific yield or specific growth rate and the *k*_*L*_*a* in the presence of antifoam, although the antifoams had affected the *k*_*L*_*a* at different concentrations. Moreover, the antifoams did not affect the dissolved oxygen concentration of the cultures. A comparison of the amount of GFP retained in the cell by flow cytometry with that in the culture medium by fluorimetry suggested that addition of Antifoam A, Antifoam C or J673A increased the specific yield of GFP by increasing the proportion secreted into the medium.

We also compared the effects of antifoams upon a membrane protein, the human adenosine _2a_ receptor (hA_2a_R). The optimum antifoam concentrations from the GFP study were added to shake flask cultures of *P. pastoris* producing this therapeutically relevant G protein-coupled receptor (GPCR). While at higher concentrations, the antifoams had been beneficial to the production of GFP, we found that the reverse was true for hA_2a_R production, and the yield of protein as determined by radioligand binding assays was lower than the controls (unpublished data).

In the last 15 years, 80% of all recombinant genes reported in the literature were expressed in either *Escherichia coli* or *P. pastoris*[36]. To date, only our study has examined the effects of antifoaming agents upon recombinant protein production by *P. pastoris* in detail and attempted to determine the mechanisms of action. The question of toxicity effects of the antifoams was not raised in any of the articles reviewed and would be useful for those using fermentation for drug production. Additionally, PEG was the most frequently investigated antifoam, possibly due to its routine use in protoplast fusion and in increasing membrane permeability to aid transformation of cells[34, 37]. Our Bartsch foaming test data has demonstrated that PEGs are not the most effective defoaming agent[32]. Many other types and compositions are commonly used in bioprocesses to reduce foaming, and the current research covers a relatively small area of research into the influence of these agents upon recombinant protein production. Of the studies that observed effects to the cells and proteins produced in the presence of antifoams, few attempted to explain the possible mechanisms of action for the findings. A summary of the findings are shown in Table 1.

**Table 1:** Summary of the biological effects of antifoam addition to bioprocesses.

Organism	Vessel	Antifoam composition	Effect on recombinant protein yield	Effect on growth rate of cells	Other observations/ mechanisms	References
		S184 (Liquid single component: silicone oil)	Reduces specific activity (mUg^-1^ dry cell mass)	No change below 250 ppm. No data reported above 250 ppm	OTR reduced in early stages of cultivation	[19]
*Eschericia coli* K-12 producing β-galactosidase fusion protein	Bioreactor (2 L and 60 L working volume)	SLM54474 (Liquid single component; polypropylene glycol	Reduces specific activity	Decreases with increasing concentration	Minimal effect on OTR and *k*_*L*_*a*	[19]
		VP1133 (Liquid single component; silicone oil/polypropylene glycol mixture)	Increases volumetric activity (mU)	No change below 250 ppm. No data reported above 250 ppm	OTR reduced in early stages of cultivation	[19]
		SE9 (Aqueous emulsion of S184 silicone oil)	Increases volumetric activity (mU)	Low µ at 555 ppm. High µ at 5000 ppm	OTR reduced in early stages of cultivation	[19]

*Geobacillus thermoleovorans* secreting α-amylase	Shake flasks	PEG8000 (Liquid single component; polyethylene glycol)	Increases α-amylase titre (UmL^-1^ culture medium) at 0.5%	No effect	Increased membrane permeability hypothesized	[34]
*Bacillus subtillis* secreting α-amylase	Bioreactor	PEG600 (Liquid single component; polyethylene glycol)	Increases productivity (U mL^-1^ h^-1^) by a factor of 1.5 at 20%	Not reported	Cells become “more hydrophilic” as measured by aqueous two-phase partition	[33]
*Bacillus amyloliquefaciens* secreting α-amylase	Bioreactor	PEG600 (Liquid single component; polyethylene glycol)	Reduces productivity by a factor of 2 at 20%	Not reported	Cells become “more hydrophobic” as measured by aqueous two-phase partition	[33]
*Schizosaccharomyces pombe* secreting human transferrin	Not clearly defined; probably shake flasks	PEG8000 (Liquid single component; polyethylene glycol)	Increases titre at 0.1%	Growth defect at 1%	None	[35]

*Saccharomyces cerevisiae* secreting Fc fusion protein	Shake flasks	Sigma Antifoam C (Aqueous emulsion; 30% emulsion of silicone polymer)	Decreased yield above 1%	No effect up to 8%	None	[10]
		Struktol SB2121 (Liquid single component; polyalkylene glycol)	Decreased yield above 1%	Decreased cell numbers with increasing concentration 0% to 8% measured by optical density	None	[10]

*Pichia pastoris* secreting Fc fusion protein	Shake flasks	Struktol J673A (Hydrophobic solid dispersed in carrier oil; alkoxylated fatty acid ester on a vegetable base	Decreased yield above 1%	Increasing cell numbers with increasing concentrations 0% to 8% measured by optical density	None	[10]

		Sigma Antifoam A (Aqueous emulsion; 30% emulsion of silicone polymer)	0.6% - 1% increases total yield	At 0.6% µ = 0.12 h^-1^ compared to control 0.13 h^-1^	0.6% increases secretion and retention of GFP	[32], unpublished data
		Sigma Antifoam C (Aqueous emulsion; 30% emulsion of silicone polymer)	0.6% - 1% increases total yield	At 0.8% µ = 0.09 h^-1^ compared to control 0.13 h^-1^	0.8% increases proportion of GFP secreted and doubles secretion compared to control	[32], unpublished data
*Pichia pastoris* secreting green fluorescent protein (GFP)	100 mL shake flask containing 20 mL culture	Struktol J673A (Hydrophobic solid dispersed in carrier oil; alkoxylated fatty acid ester on a vegetable base	0.4% to 1% increases total yield; 1% almost doubles yield	At 1% µ = 0.19 h^-1^ compared to control 0.13 h^-1^	0.8% increases proportion of GFP secreted and doubles secretion compared to control	[32], unpublished data
		Fluka P2000 (Liquid single component; polypropylene glycol)	0.6% to 1% increases total yield; 1% doubles total yield	At 1% µ = 0.15 h^-1^ compared to control 0.13 h^-1^	0.6% increases proportion of GFP retained	[32], unpublished data
		Struktol SB2121 (Liquid single component; polyalkylene glycol)	0.4% to 1% increases total yield; 1% doubles total yield	At 1% µ = 0.14 h^-1^ compared to control 0.13 h^-1^	0.6% increases proportion of GFP retained	[32], unpublished data

## How do antifoams interact with cells and proteins in bioprocesses?

We have observed that antifoams can affect the growth of yeast cells, and similar observations have also been made for bacteria[10, 19]. Increased growth rates of cultures have been found to lead to increased productivity[38, 39] which is true for our observations for 0.6% Antifoam A, 1% J673A, 1% P2000 and 1% SB2121 cultures which grew at similar or higher growth rates than the control cultures and produced a higher yield of GFP. However, some studies aiming to control growth rates in order to improve specific productivity (q_p_) have found that maximal specific growth rates did not relate to maximal specific productivity[40–43]. It has also been found that high levels of protein expression may lead to a reduction in specific growth rate[44]. This could explain the results we obtained for Antifoam C at 0.8% which grew at a lower growth rate than the control but still produced a higher yield of protein and for the results obtained by Koch *et al* who found that increasing concentrations of SLM54474 decreased growth rate but increased enzyme titres[19]. It seems that the relationship between growth rate and productivity varies depending upon the specific parameters of the cultures.

Antifoams are known to affect the *k*_*L*_*a* of a system, but our data suggested that this may not itself be enough to influence the overall DO level in the medium. We concluded that our observations were not due to the effect of antifoams upon oxygen transfer. Although the study by Koch illustrated that antifoams did affect oxygen transfer rates and *k*_*L*_*a*, the data was not used to explain the effects of the antifoams upon the organisms themselves.

In the case of PEG influencing secretion of proteins, studies suggested that the PEG altered the state of bacterial and yeast cell membranes allowing improved secretion of recombinant protein[34, 35]. This is consistent with an earlier study which suggested that antifoams can affect cell permeability in yeast by perturbing sterol biosynthesis which then alters the permeability of the membrane[45]. Yeast plasma membranes contain polar lipids such as glycerophospholipds and sphingolipids. Non-polar lipids consist of free fatty acids, diacylglycerols, triacylglycerols, sterols and steryl esters[46]. Ergosterol is a major component of yeast plasma membranes[47–49] and helps to maintain the structure of the membrane[48] as sterols are rigid hydrophobic molecules with a polar hydroxyl group[50]. Membrane fluidity is important for nutrient uptake and exchange of substrates[50], and affects the movement and activity of membrane proteins and insertion sites[51]. Fatty acids and sterols affect the fluidity of the membrane[51]. Combining flow cytometry and fluorimetry data in our study showed that antifoams can influence the amount of GFP retained inside the yeast cell as well as the amount secreted into the medium. Antifoam A, Antifoam C and J673A enhanced the GFP secreted compared to 0% antifoam suggesting that the increase in total yield observed could be due to this secretion effect[32]. Preliminary analysis of electrospray mass spectrometry data suggested changes in relative phosphatidylcholine composition in 1% P2000 samples and changes in relative phosphatidylinositol composition for all antifoam-containing cultures compared to controls (unpublished data). It has also recently been shown that alterations in the ergosterol biosynthesis pathway of *P. pastoris* have been linked with increases in recombinant protein secretion and that surfactants may affect the membrane fluidity also leading to a greater amount of secreted protein[52].

It is also worthwhile to note that there is evidence to suggest that vegetable oils may be metabolized as a carbon source[31], but there is no information regarding the ability of yeast to metabolize the other agents such as silicone polymers or polyalkylene glycols. It could be possible that some organisms are able to utilize antifoam agents and this enhances their ability to grow and produce protein.

## Conclusion

The biological effects of antifoams are poorly understood and this is in part due to the range of types available and the lack of information regarding their compositions being available from the manufacturers. Antifoams have commonly been added to bioprocesses without full knowledge of their possible effects, but as an additive, these effects should be assessed. Published studies have demonstrated that each antifoam not only destroys foam with a range of effectiveness, but may also affect the cells and the proteins themselves. The concentration and type of antifoam required to alleviate foam should therefore be balanced with the possible effects it could have upon the process. Consequently, screening for optimum conditions is required. Our study and that of Koch *et al* demonstrated that higher concentrations of antifoam than would normally be used can benefit the process, however it has also been suggested that antifoams could damage fermentation equipment[13], and they are known to foul membranes in downstream processing[53]; therefore consideration of the whole process must be taken. In summary, these investigations have illustrated that antifoams could increase the productivity of a process or hinder it. It is not likely that the precise mechanisms of antifoams action will be easily understood, especially as a combination of factors may have led to the effects upon protein yields. For these reasons, it is important to thoroughly evaluate the effects of antifoam addition to fermentation cultures on both a small and large scale on a case-by-case basis.
